# Croatian genetic heritage: Y-chromosome story

**DOI:** 10.3325/cmj.2011.52.225

**Published:** 2011-06

**Authors:** Dragan Primorac, Damir Marjanović, Pavao Rudan, Richard Villems, Peter A. Underhill

**Affiliations:** 1 University of Split, Medical School, Split, Croatia; 2 University of Osijek, Medical School, Osijek, Croatia; 3 Eberly College of Science, Penn State University, University Park, Pa, USA; 4 University of New Haven, New Haven, Conn, USA; 5 Institute for Genetic Engineering and Biotechnology, Sarajevo, Bosnia and Herzegovina; 6 Genos, Zagreb, Croatia; 7 Institute for Anthropology, Zagreb, Croatia; 8 Croatian Academy of Sciences and Arts, Zagreb, Croatia; 9 Institute of Molecular and Cell Biology, University of Tartu and Estonian Biocentre, Tartu, Estonia; 10 Department of Genetics, Stanford University, School of Medicine, Palo Alto, Calif, USA

## Abstract

The aim of this article is to offer a concise interpretation of the scientific data about the topic of Croatian genetic heritage that was obtained over the past 10 years. We made a short overview of previously published articles by our and other groups, based mostly on Y-chromosome results. The data demonstrate that Croatian human population, as almost any other European population, represents remarkable genetic mixture. More than 3/4 of the contemporary Croatian men are most probably the offspring of Old Europeans who came here before and after the Last Glacial Maximum. The rest of the population is the offspring of the people who were arriving in this part of Europe through the southeastern route in the last 10 000 years, mostly during the neolithization process. We believe that the latest discoveries made with the techniques for whole-genome typing using the array technology, will help us understand the structure of Croatian population in more detail, as well as the aspects of its demographic history.

## Prologue

The origin of the ancestors of modern Europeans has been discussed by archaeologists, anthropologists, linguists, geneticists, and other scientists. Some archeologists use the term “Old Europe” to describe the widespread pre-Indo-European Neolithic culture in Europe, particularly the Balkans, and they regard it synonymous to the terms Neolithic Europe and Pre-Indo-Europe (1). But, if the Neolithic people are referred to as Old Europeans, how should we describe the people who had been living all over Europe even before the last Ice Age? The results of the latest genetic analyses based on Y-chromosome polymorphisms support the existence of two main branches of European men: ‘Old Europeans,’ who include parental lineages that had already existed in Europe before the last glacial maximum (LGM) and ‘Early Farmers,’ who had arrived from west Asia as either hunter gatherers during the early post-glacial period or as pioneer farmers from the Fertile Crescent, the region extending from the eastern Mediterranean coast to the Persian Gulf and the Tigris and Euphrates valleys (2). In addition, it is possible that migrations to Europe of both hunters and farmers could have been contemporaneous during the early Neolithic transition. Evidently, the term ‘Old Europeans’ as used by molecular anthropologists is more open and extensive than when it is used outside of the genetic context.

Here we address the contribution of these two branches to the recent Croatian human gene pool, in other words we examine the origins of the modern Croatians. Similar questions were addressed 10 years ago in a study on the origin of the Europeans (3). The article by Semino et al published in Science in 2000 (4) stimulated a heated discussion on the Croatian genetic heritage. It is fascinating that a single scientific report is able to simultaneously trigger an avalanche of new scientific ideas and approaches and create a platform for so many non-scientific debates.

Starting from the fact that the human genome is an updated record of its own evolution and history (5), we present an overview of scientific data about the Croatian genetic heritage that have been collected over the past 10 years.

Population genetics is a potentially useful tool in the examination of the past human migrations (5), especially if we understand that current patterns of genetic variation are the key to gaining an insight into past population processes (4,6-8). Also, genetic polymorphisms can nowadays be used to infer the population of origin of an individual (9-11). The most interesting and useful information is provided through the analysis of two uniparental markers within our genome: the Y-chromosome and mitochondrial DNA (mtDNA).

Several features make Y-chromosome a useful and interesting phylogenetic tool. It is haploid and male-specific, passed from father to son, and since it is for over 95% of its length excluded from meiotic recombination, changes in it occur only by mutation. It also displays an extraordinary amount and variety of different classes of genetic markers (12). This accumulation of sequence variation during the lineal life spans of Y-haplotypic systems provides a powerful resource for the recovery of genetic prehistory (6). If we imagine human population as one woman and one man, this couple carries only one Y chromosome. Therefore, effective population size for the Y-chromosome is expected to be one-quarter of that for any autosome and one-third of that for the X-chromosome. In addition, almost 70% of modern societies practice patrilocality, which means that men tend to live closer to their birthplaces than women, so Y-chromosome is expected to show greater geographical clustering than other population markers (13). Several years ago, a comprehensive haplogroup tree was constructed for the human Y-chromosome by genotyping most of the known polymorphisms. Initially, this tree showed the relationships among 153 haplogroups based on 243 binary markers (14). Several years later, a revised tree was published, which already contained 311 distinct haplogroups and incorporated approximately 600 binary markers (15). These numbers continue to expand. Before the construction of haplogroup tree, partial Eu haplotypes were used (4).

Similar features could be recognized for mtDNA – phylogenetic relationships among mitochondrial haplotypes in a sample reflect the maternal genealogical relationships among the sampled individuals and phylogenetic tree of global human mitochondrial DNA variation, based on both coding and control region mutations (16-18).

Recent identification of a large number of informative biallelic markers in non-recombining region of the Y-chromosome (NRY) and mtDNA markers has already significantly contributed to the understanding of European prehistory and history. In this overview, we deal with only a few of them. This selection, unlike that in some previously published studies (19), is based mostly on Y-chromosome results.

## European Y-chromosome story

A few years ago, Wiik published a very interesting and informative study under the attractive title – Where Did European Man Come From (2). Its notable size (50 pages) and information quality (number of the references, maps, and other figures) make this article worthy of a respectable book chapter. As a starting point for our ‘Croatian genetic heritage’ journey, we made a short overview of this article. We also updated phylogeographic diversification patterns within Y chromosome haplogroups E and R and their reappraisals (20-22).

According to Wiik, almost all European men belong to 11 Y-haplogroups (in alphabetical order: E3b, G, I1a, I1b1-P37, I1b2-M223, J2, N3, R1a, R1b, E3a, N2) (2). Male-mediated migrations as characterized by Y-haplogroup classification can be presented through several stages. The first one, which was divided in 10 phases, represents the earliest movement of the European man’s ancestors. Nine of them predate the LGM.

Wiik’s story started 50 000 years ago, when all the ancestors of European man still lived in northeastern Africa within one single clan. Twenty thousand years later, the first specimens of *Homo sapiens* (clan R1) reached European soil (the steppe area between the Ural mountains and the Caspian Sea) via Euroasia. Approximately 5000 years later, the first men settled at the Iberian Peninsula and the Atlantic Coast (clan R1b). Almost at the same time, a new clan arrived to the Balkan area from the Middle East, through Anatolia. This small group was responsible for bringing an important genetic entity for our story – haplogroup I. Very soon after this first arrival, approximately 20 000-13 000 years ago (4), human populations across Eurasia experienced the Last Ice Age. This event significantly reduced the inhabitable territory for human population. European men’s ancestors were forced to survive within 4 large refugia located in Ukraine, Iberia, the Balkans, and possibly Siberia. These isolated locations minimized gene flow and enhanced the process of genetic drift in forming distinct genetic patterns in terms of allele frequencies and the appearance of new regionally-specific mutations. These incubators of genetic diversification during the LGM later became source regions for the recolonization of Europe during the post-glacial period and Holocene (10 000 years ago). Simultaneously with these post-LGM migrations, new clans from the Middle and Near East and Anatolia arrived to Greece and spread out along the Mediterranean coasts. They brought with them domestication of wild animals and plants, and shared their knowledge with the autochthonous population. This was the beginning of agriculture and cattle breeding in Europe.

According to this source, it could be concluded that Y-chromosome background may cluster all European men within two main branches: ‘Old Europeans’ with the parental lineages Hg I, possibly Hg G and Hg N who had already been present in Europe before the LGM and who survived this period in 4 European refugia, and ‘Early Farmers’ (Hg E3b, Hg J2, some subclades of Hg G) who had still been on ‘summer vacation’ in Asia and Africa during the LGM and arrived during the neolithization of Europe.

However, while the Wiik study does a reasonably good job summarizing the earlier literature about Europe, it has recently become clearer that the previously established model (4) stating that Hg R dates from the Paleolithic should be revised. The latest studies in this field (20,21) suggest that Hg R membership, be it R1a-M17 or R1b-M269, in Europe is a more recent (post-LGM) event (about ≤15 000 years ago). According to those recent findings, it is possible that these Hg R lineages began to spread from Western Asia into Europe soon after the ice sheets began to retract but before the arrival of farming in southeast Europe and Crete about 9000 years ago. So, this model suggests that 15 000 -10 000 years ago, Europe was inhabited by Mesolithic people, some being indigenous Hg I and some being post-glacial intrusive Hg R from West-Asia. Then, pioneering agriculturalists came from the Fertile Crescent and acquainted the local foragers with farming.

Wiik (2) believes the whole continent could be observed in terms of 8 separate regions regarding Y-chromosome background and the existing European language groups. One of these is particularly interesting to our discussion on Croatian Y-chromosome heritage – the Balkans. Wiik describes several interesting Y chromosome gradients in this area: a) north-south gradient of haplogroup Hg R1b in the north of the Balkans, which is probably connected with spreading of the West-European or Iberian men to the Balkans, b) north-south gradient of haplogroup R1a, which is probably connected with the migrations from the Eurasian area, c) very high frequency of haplogroup I1b1-P37 in the Western Balkans that diminishes in all directions, which is probably correlated with the existence of a glacial era refugium and post-LGM recolonization by Old Europeans from that area d) a south-north gradient of haplogroup E3b and similar but weaker gradient of haplogroup J2, which is probably connected with migration of Early Farmers. However, according to Battaglia et al (22), almost all European Hg E3b are actually derived for the V13 mutation which seems to have arisen in the Balkans from a M78* molecular ancestor. Haplogroup E-V13 does not seem to have come from the Near East but,according to King et al (23), its presence is much more likely a consequence of Greek colonization. Also, there is no V13 in the areas of the Fertile Crescent where farming originated,. So, contrary to what was proposed by Wiik (2), the Hg E3 in Europe probably arose locally as a Balkan specific lineage, probably not earlier than 8000-10 000 years ago and then experienced demographic growth when farming took hold.

It is indicative that Wiik recognized the specificity of the Croatian population, which was elaborated in a separate section. He concluded that while the Hg R1a men arrived to Croatia mainly from the Ukrainian refugium, the Hg R1b men arrived from the Iberian refugium. The West Balkan peak area of haplogroup I1b1-P37 is in central Croatia, which could be described as the center of Balkan refugium and the epicenter of the post-LGM European decolonization from this area. I1b1-P37 diverged from Hg I possibly during that decolonization and HgI arrived to this area approximately 25 000 years ago from the Middle East.

The most recent data (20,21) describing the R1b-M412 lineages do not support Wiik’s proposition that R1b in Croatia came from Iberia. If that was correct, the current population in Croatia would have the very frequent M412 mutation. However, Myres (20) clearly showed that Balkan R1b lacked this mutation and had the L23 precursor variety. It is clear that Balkan R1b most likely did not come to Croatia from Iberia but from some unspecified place in the West Asia, perhaps via the Levant (Lebanon), but independently of farmers. Furthermore, it is possible that R1a-M17 lineages in Croatia were introduced from the northern part of Eastern Europe by the widespread copper and bronze age Corded Ware cultures (5200-4300 years ago) down through the more recent Slavic expansions. Archeologists agree that the Corded Ware tradition spread from Central Europe eastwards to the Volga.

**Figure 1:**
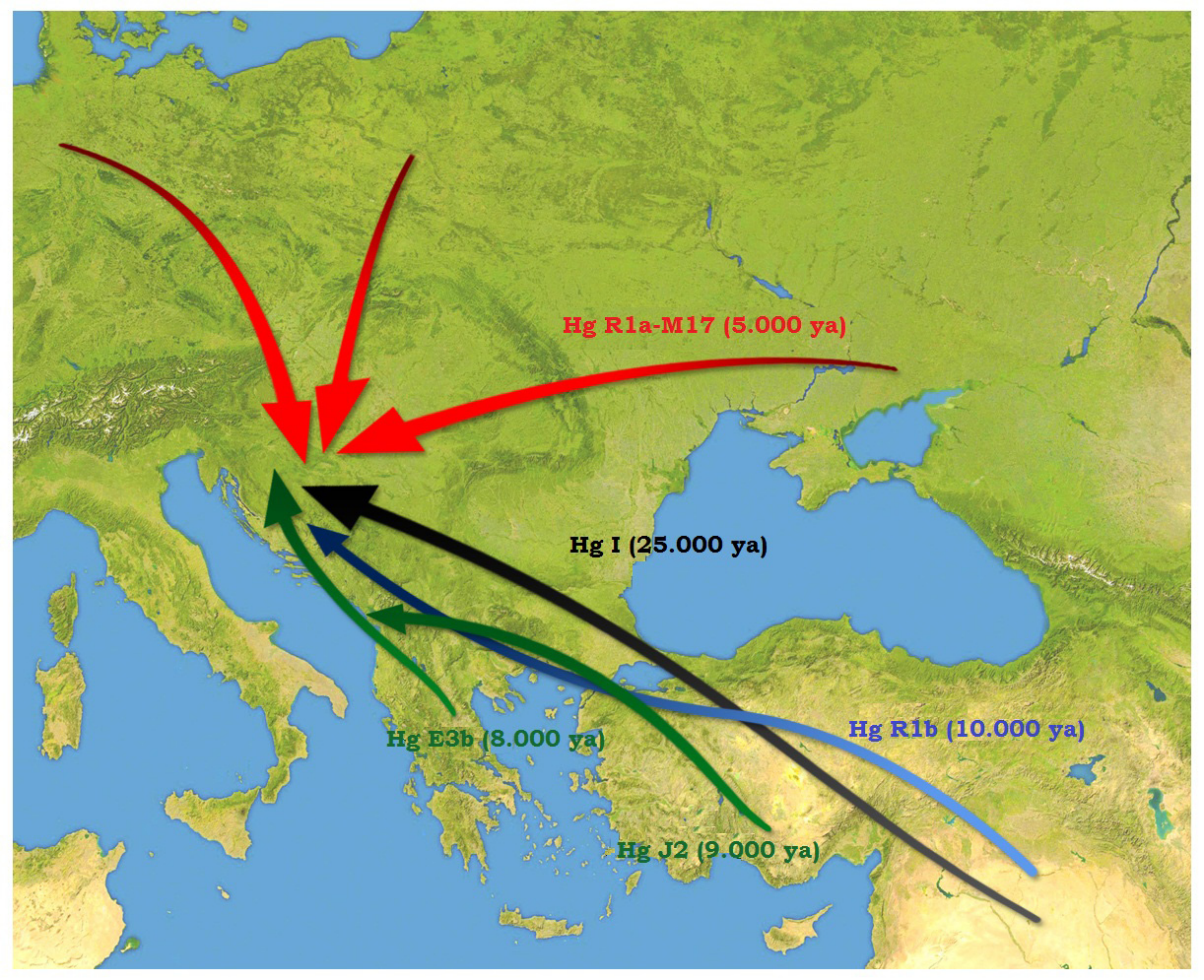
Proposed migration routes for the main observed haplogroups for the present Croatian area: black (HgI) – pre-Last Glacial Maximum (LGM) migrations from the Middle East; blue – post-LGM migrations from West Asia (trough the Levant); red (R1a-M17) – post-LGM migrations from Ukrainian LGM refugium and Germany/Poland region; dark green (J2) – Early Farmers from the Near East; light green (E3b) – Balkan-specific lineage spread by Greek colonization. The migration routes are drawn according to results published by Semino et al (4) and several other authors (2,20,21).

We are going to focus on these conclusions, but by examining several important earlier reports, particularly that by Semino et al (4), which were on the reference list of the Wiik (2) and some more recently published studies (Figure 1) (20,21).

## Starting point

As indicated earlier, nobody could predict that a single scientific report from the field of molecular anthropology and population genetics (4) could trigger such an intensive discussion within Croatian scientific and non-scientific community as did the collaborative international study by Ornella Semino of the University of Pavia, Italy and Peter A. Underhill of Stanford University, California, USA. This Y-chromosome study of 1007 men from 25 different regions in Europe and the Middle East proposed 3 waves of migrations to Europe: approximately 40 000, 22 000, and 9000 years ago. The authors suggested that more than 80% of European men inherited Y-DNA from Paleolithic ancestors who had lived in Europe 25 000 to 40 000 years ago, ie, Old Europeans according to Wiik (2). The other 20% inherited their Y-chromosomes from Neolithic farmers who had arrived to Europe 9000 to 10 000 years ago. Almost immediately after its publishing in Science, all co-authors, especially the one from Croatia (Dragan Primorac), became famous and appeared in numerous newspapers articles and TV shows. What was even more interesting, many persons, with various scientific backgrounds, interpreted the meaning of the results to the public. Some of those explanations were rational, some of them completely wrong, and some of them were nothing but entertainment.

The main goal of the study was to present the human history of Europe from a genetic perspective derived from 22 NRY binary markers. Twenty-two selected NRY binary markers were typed in 1007 men from 25 different European and Middle Eastern geographic regions. Fifty-eight of those samples originated from the Croatian population. The most important conclusion was that nearly all of the European Y-chromosomes analyzed in the study belonged to 10 lineages characterized by simple biallelic mutations. Also, a substantial portion of the European gene pool appeared to be of Upper Paleolithic origin, but was relocated after the end of the LGM, when most of Europe was repopulated (24). This was one of the pioneer articles in this field, which offered Y-chromosome molecular-genetic scenario for peopling of Europe (25). Also, these were the first results that supported the existence of several LGM refugia for Old Europeans. In addition, it recognized the male contribution to the demic diffusion of Early Farmers from the Middle East to Europe, which seems to have been more pronounced along the Mediterranean coast than in Central Europe.

**Figure 2:**
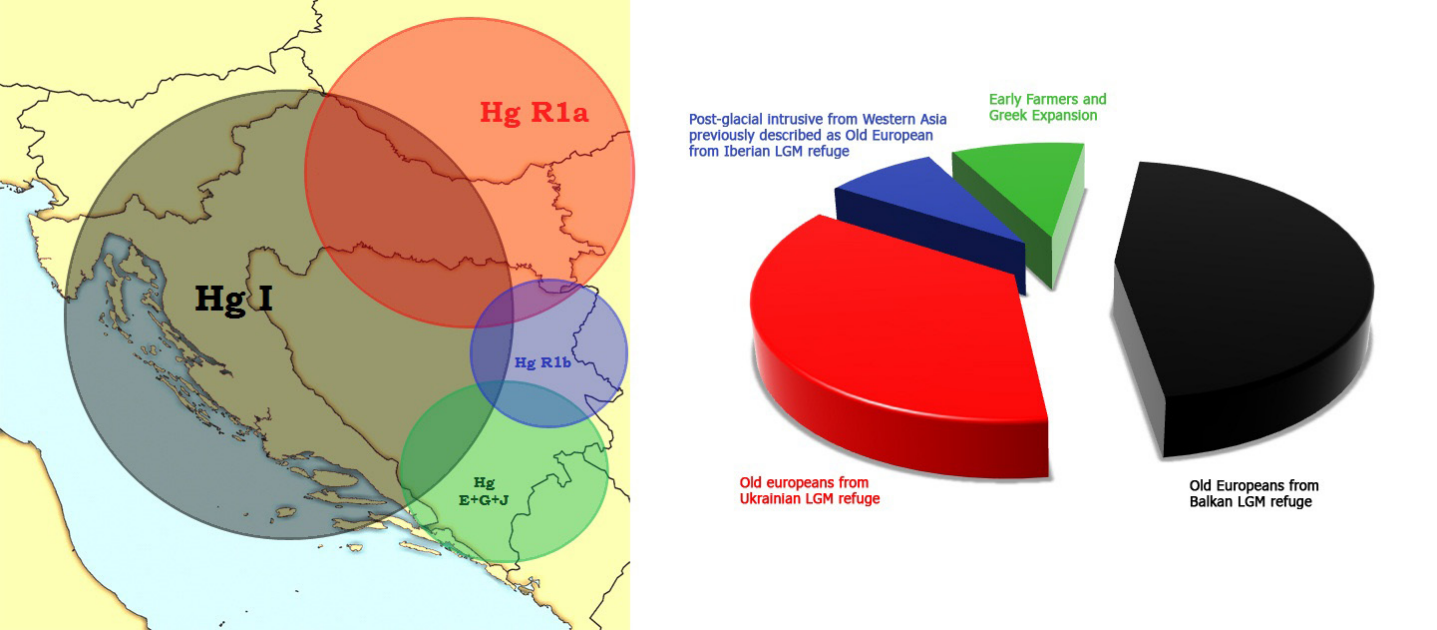
Croatian Y-chromosome population structure according to data published by Semino et al (4) Approximately 45% of the examined Croatians probably originated from the Old Europeans who mostly survived the Last Glacial Maximum (LGM) in the Western Balkan refugium. In addition, almost 30% of them came from the Ukrainian LGM refugium and 10% from postglacial intrusion from Western Asia (20,21), previously described by Semino as Old Europeans from Iberian refugium (4). The rest of the Croatian men (approximately 15%) originated from Early Farmers who brought agriculture into Europe from the Middle East.

As far as Croatian population is concerned, Semino et al found that 45% of the examined Croatian men belonged to the Eu7 haplotype (synonymous with the currently defined haplogroup I). In addition, almost 30% of them belonged to the Eu19 (haplogroup R1a1) and around 10% to the Eu18 (haplogroup R1b). The rest of the Croatian men (approximately 15%) were divided between Eu4 (E3b), Eu9 (J2a), Eu11(G), and Eu16 (LT) haplotypes (Figure 2). They also concluded that 45% of the examined Croatians probably originated from Old Europeans who mostly survived LGM in the Western Balkan refugium. After the climate had improved, this population spread north and east. The rest of them originated from the farmers who brought agriculture from the Middle East to Europe. Only 3 of the 58 examined Croatian men who were typed as Eu16, could not be specifically included in any of these 4 groups, because of a lack of suitable NRY markers at the time. That was all – nothing more and nothing less!

Croatian results offered in this work could be recognized as a remarkable starting point for the following studies.

## Logical following step

As it was expected, the Croatian scientific community, led by the scientists from the Institute for Anthropological Research, in collaboration with a respectable scientific team from Estonia, recognized the potential of the results by Semino et al. Three years later, equipped with the scientific knowledge gathered during 2000-2003 period, more discovered NRY genetic markers, new Y Chromosome Consortium (YCC) nomenclature of the Y-chromosome haplogroup model, and very efficient and usable YCC tree (14), Barać et al published an informative and detailed article about Y-chromosome heritage of Croatian population (26). Samples were obtained from 457 Croatian men, approximately 1/4 of them were from the Croatian mainland and more than 3/4 from 4 Adriatic islands. These 4 islands (Korčula, Brač, Hvar, and Krk) are one of the best studied regions in European population genetics in the last 25 years.

Almost 49% of the examined Croatian men belonged to HgI, almost 27% of them belonged to HgR1a, and less than 7% to the HgR1b. The rest (approximately 20%) were divided among haplogroups E, G, J, F, K, and P (26). Again, it was concluded that 49% of the examined Croatians probably descended from the Old Europeans who mostly survived LGM in Western Balkan refugium. Western Balkan Peninsula may have been an LGM reservoir of HgI, a starting point of an expansion that spread HgI through the neighboring populations. The fact that the second most frequent haplogroup in the mainland and island populations was R1a implies that at least some of the founding ancestral groups of Croatian population originated from Indo-European speaking populations who had possibly migrated from southern Russia 2000 BP carrying this mutation. Also, M173 mutation (R1a) was more pronounced in the northern part of Croatia than in the southern, coastal area. The observed frequencies of haplogroups E, G, and J in the Croatian sample were low, suggesting a minor genetic impact from the Early Farmers from the Middle East. This was in discordance with some of the previously published studies (27).

Shortly, this more detailed and informative study confirmed the preliminary results from the report by Semino et al (4). Despite the fact that more than of 3/4 of the examined men originated from a relatively isolated island population, this study could be recognized as one of the most informative reports to date about Croatian genetic heritage.

## About the closest neighborhood

Bosnia and Herzegovina and Croatia share very long continental borders and common history. Also, Croatians are one of the 3 constitutive ethnic groups in Bosnia and Herzegovina. Due to all these reasons, population studies in Bosnia and Herzegovina may provide interesting insights about the Croatian population.

In 2005, an international team, led by the Institute for Genetic Engineering and Biotechnology employed 28 Y-chromosome biallelic markers to analyze 256 men (90 Croats, 81 Serbs, and 85 Bosniacs) from Bosnia and Herzegovina (28). The population sample was representative of all of Bosnia and Herzegovina, since the participants originated from more than 50 different locations. Participants were attributed to an ethnic group according to the origin of their paternal grandfather. All samples were classified into haplogroups E, F, G, I, J, K and R according to the chosen Y-chromosome markers. The most commonly represented haplogroup was haplogroup I, accounting for more than 50% of the Y-chromosomes. Additional haplogroups with overall frequencies higher than 5% were haplogroups E (14.5%), R-M17 (13.7%), and J (7.1%).

The analysis showed that all 3 ethnical groups had the same haplogroups as other Europeans who had originated from different glacial refugium areas of Europe (I-M170, R-M17 and RM269 from Balkan, Ukrainian refugia, respectively), and as Europeans who had originated from Africa (E-SRY4064) and the Middle East (J-12f2) and arrived to Europe through the prolonged gene flow from the Middle East (29).

**Figure 3:**
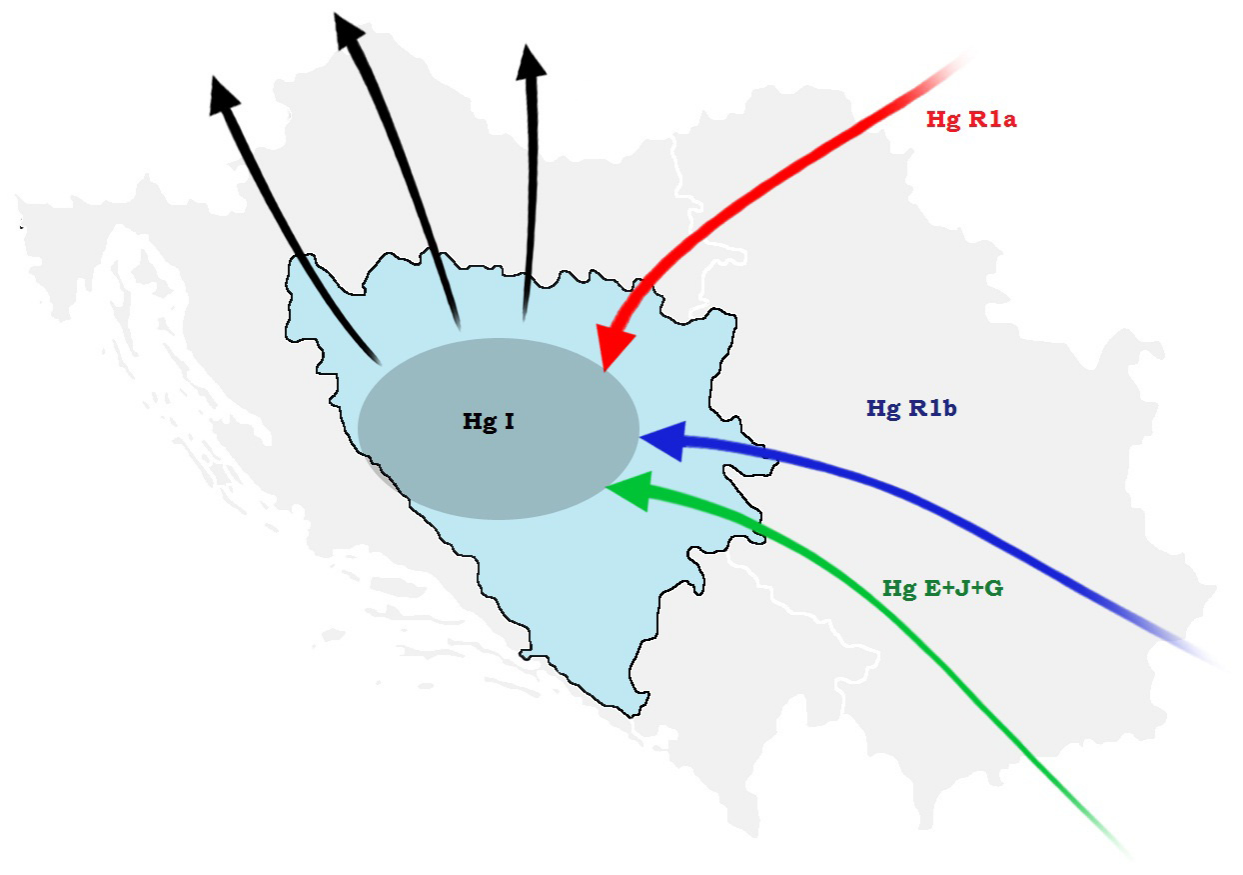
Interpretation of the Bosnia and Herzegovina Y-chromosome tree by Marjanović et al (28), according to which the territories of today’s Croatia and Bosnia and Herzegovina were probably part of the Balkan Last Glacial Maximum refugium.

Taking into account the Paleolithic origin of the P37 mutation in this region of the Balkans (29) and its extremely high frequency (more than 50%), it is possible that the post-LGM expansion of a population with a high frequency of I-P37 from one of the refugia in the Balkans played a major role in the peopling of Bosnia and Herzegovina and the surrounding areas, including Croatia. These results suggest that today’s Croatia and Bosnia and Herzegovina were probably a part of the Balkans LGM refugium. Joint frequencies of the haplogroups J and G are more than 9%, suggesting that 9% of Bosnia and Herzegovina men originated from the Early Farmers. According to the previously mentioned data (22), E-V13 in the Balkans (14% in Bosnia and Herzegovina) is a local marker that had existed prior to farming at a low frequency but increased with the spread of farming (22). These results suggest that male Bosnia and Herzegovina population’s gene pool is more influenced by the Neolithic and post-Neolithic migrations than the Croatian gene pool. On the other hand, relatively low frequencies of R1a and R1b haplogroups in Bosnia and Herzegovina male populations show a smaller portion of genes from the Ukrainian LGM refugium than in the Croatian population (Figure 3).

The frequency of sub-haplogroup I-P37 observed in the Bosnia and Herzegovina Croats is high among all 3 Bosnia and Herzegovina ethnic groups and could be partially attributed to genetic drift, but still is the highest detected frequency for some populations. In contrast, Neolithic and post-Neolithic gene flow appears to have played a marginal role in the Bosnia and Herzegovina Croats. Finally, the migratory processes from Central Asia and Eastern Europe – marked by haplogroup R-M17 – similarly influenced the 3 major ethnic groups of the modern Bosnia and Herzegovina

## Additional thoughts on Old Europeans and Early Farmers in Croatia

The article by Battaglia et al focused on the analysis of the relationship between Old Europeans and Early Farmers (22), as well as the article by Rootsi et al (22,30). Both studies (22,30) included Croatian and Bosnian populations and scientists from the two countries.

Regarding the Old Europeans, additional analysis of more than 1000 Hg I Y chromosomes from 60 population samples revealed several subclades in Europe, with divergent geographic distributions (30). Authors suggested that haplogroup I provided an excellent record of pre-LGM differentiation followed by geographic contraction, isolation, and subsequent post-LGM expansions and spreading. Occurrence of I1a in Scandinavia is consistent with a post-LGM recolonization of northwestern Europe from Franco-Cantabria. The expansion of I1b* in the wider Adriatic area suggested demographic processes that started from a refugium located in that region, whereas, I1c covers a considerable part of Europe, with the highest frequencies in northwestern Europe. It is suggested that haplogroup I originated from a pre-LGM pool of Europeans (28,000-23 000 years ago). Also, it appears that I1a, I1b, and I1c diverged from I*, possibly during the post-LGM recolonization of Europe. Regarding the Old Europeans in this area, high short tandem repeat diversity within I1b* lineages in Bosnia and Herzegovina and Croatia supports the view that the P37 short nucleotide polymorphism may have been present in the Balkan area before the LGM. This implies that the territories of these two countries were probably very attractive for “summer vacation” during the LGM.

The article by Battaglia also included the analysis of the Croatian gene pool (22). More than 1200 unrelated male individuals from 17 populations were included into this study and special attention was paid to several subclades within haplogroups E, J, and I. It was found that Old Europeans from the Balkans were the first to adopt farming when it was introduced by Early Farmers from the Near East and spread this way of life throughout the Adriatic area and transmitted the Neolithic culture to other Old European populations. This genetic evidence, together with some other studies performed only on Y-STR (31), supports the model of cultural diffusion.

## Conclusion

Population genetics could sometimes result in very interesting predictions on different groups within populations (32,33). Molecular genetic analysis of the genetic pool of modern Croatian male population confirmed extraordinary heterogeneity and complexity of this population and confirmed that there was a high degree of mixing of the newly arrived settlers with the indigenous populations that had already been present in the region. As illustrated in this review, earlier research offered a genetic scenario for the most important migration episodes that strongly influenced the peopling process of the territory of modern Croatia. Most of those studies investigated the ancestral genetic impact of Old Europeans and Early Farmers on Croatians. They proved that Croatian population, as almost any other European population, represents a remarkable genetic mixture.

Previous studies (2,4,26,28) clearly concluded that most of Croatian men (‘owners’ of HgI) descended from the people who settled in Europe approximately 25 000 years ago and survived the LGM in the Western Balkans refugium. Since the latest studies (20,21) proposed a completely new background of R1b migration and since there are 27% of the R1a holders in Croatia, it could be concluded that more than 3/4 of the contemporary Croatian men are most probably the offspring of Old Europeans who came here before and after the LGM. The rest of the population are the offspring of people who arrived in this part of Europe trough the southeastern route, in the last 10 000 years, mostly during the Neolithization process. We are certain that the latest discoveries with the techniques for whole-genome typing, using the array technology, will help us understand the structure of Croatian population in more detail, as well as the aspects of its demographic history. This approach, which we intend to use in future investigations of the human population in this region, has already yielded many interesting results in the analysis of different populations all over the world (34-36).
